# Under pressure and overlooked: the impact of COVID-19 on teachers in NSW public schools

**DOI:** 10.1007/s13384-022-00518-3

**Published:** 2022-04-07

**Authors:** Leanne Fray, Felicia Jaremus, Jennifer Gore, Andrew Miller, Jess Harris

**Affiliations:** Teachers and Teaching Research Centre, School of Education, The University of Newcastle, CT Building, University Drive, Callaghan, NSW 2308 Australia

**Keywords:** COVID-19, Teacher, Wellbeing, Australia, Morale, Self-efficacy

## Abstract

The COVID-19 pandemic has put unprecedented pressure on teachers around the world, raising significant concerns about their workload and wellbeing. Our comparison of 2019 (pre-pandemic) and 2020 (first year of the pandemic) survey data (*n* = 362) from teachers in New South Wales, Australia, demonstrates that their morale and efficacy declined significantly during COVID-19, even with the relatively short period of school closure (8 weeks) during 2020. Interviews with teachers and school leaders (*n* = 18) reinforced these findings and highlighted the depth to which teachers felt dispensable and unappreciated, despite working incredibly hard for their students. The pressure to adapt to online teaching and learning, in trying circumstances, also challenged their confidence in their teaching. We argue that practical and emotional support for teachers both during periods of remote learning and upon students’ return to the classroom is essential to support teacher’s wellbeing and a robust teaching workforce into the future.

## Introduction

The COVID-19 pandemic has disrupted life on an unprecedented scale, globally. In efforts to contain the spread of the virus, governments around the world closed entire schooling systems, affecting more than 90% of the global student population during 2020 alone (Psacharopoulos et al., 2020; UNESCO, 2020; United Nations, 2020). In New South Wales (NSW), Australia, where this study was conducted, ‘non-essential’ workers were required to stay home and keep their children home from school, where possible, for a (relatively short) period of approximately 8 weeks from March 2020. In response, teachers rapidly developed approaches for students to engage in ‘learning from home’. Most teachers moved to online modes of teaching in a matter of days with limited external support (Clinton, 2020; Norman, 2020). The Delta variant of COVID-19 caused a second state-wide lockdown in 2021, again forcing students and teachers into remote learning, with most students and teachers in NSW spending up to 14 additional weeks in remote learning. These disruptions to schooling remain substantially less severe than in other nations where there are more than 168 million children who have missed more than one year of schooling (UNICEF, 2021).

During the early weeks of school closures in 2020, admiration and respect for teachers increased, particularly as parents and carers faced the challenging task of teaching their own children from home (Doyle, 2020; Duffy & Kent, 2020). Stories honouring the work of teachers were shared—delivering learning materials to homes, making daily phone calls and providing back up for children and their families during difficult and demanding times. The status of teachers appeared to lift as the necessity and complexity of their work was made more visible to parents and the wider community (Heffernan et al., 2021). While classified as essential workers, teachers were viewed with a level of appreciation similar to nurses and doctors on the ‘frontline’ of the pandemic (Victoria, 2020).

Sadly, this apparent lift in status did not last long in media reports. Teachers have long been undervalued, even described as a part of a “battered profession” (Dinham, 2013, p. 98) in which they feel demoralised and unappreciated (Mackenzie, 2007; Stroud, 2018), sometimes leading to burnout (Whiteoak, 2020). Indeed, prior to COVID, teaching was recognised internationally as a highly stressful occupation (Dabrowski, 2020; The NEiTA Foundation & ACE, 2021), with more than fifty percent of teachers in some locations already reporting they were unable to “keep work stress at an acceptable level” (NSW Public Service Commission, 2019, p. 4) and already at high risk of burnout (García-Carmona et al., 2019). Increased scrutiny from governments (Gallant & Riley, 2017), heightened expectations from the community (Alhamdan et al., 2014), declining pay levels compared to other professions (Rajendra, 2021) and diminishing access to centralised support (Manuel et al., 2018) have resulted in high levels of teacher attrition with ‘critical’ teacher shortages predicted both in Australia (Henebery, 2020) and internationally (Holmqvist, 2019).

Given this precarious state of affairs, it is important to examine how teachers fared during COVID, a global crisis that arguably amplified demands on the teaching workforce. While early anecdotal reports within the media suggested teachers were mentally and physically exhausted (Collie & Martin, 2020; Forster, 2020), rigorous research that probes teachers’ lived experiences during COVID is needed, particularly to guide future planning efforts. To date, the impact of the pandemic on the wellbeing and development of *students* has, understandably, taken priority in most COVID research (e.g. Hoffman & Miller, 2020; Kuhfeld et al., 2020). However, given the impact of teachers’ work in shaping futures for all young people, it is vitally important that we also examine the impact on teachers.

Aside from a small number of peer-reviewed publications, extant research largely consists of commentaries, grey literature reports and studies that are not yet peer reviewed. Available literature signals a multifaceted array of challenges impacting teachers’ lives since the beginning of the pandemic, including shifting government advice, intensified workloads and increased psychological challenges for both student and teacher wellbeing. One quantitative study of more than 7000 teachers in the US found the move to remote teaching caused a decline in feelings of success (Kraft et al., 2020), while a mixed-method study involving 151 teachers in the US found remote teaching increased emotional exhaustion (Chan et al., 2021). Studies from Switzerland (Hascher et al., 2021) and Portugal (Alves et al., 2021) report reduced teacher wellbeing while, in Australia, reports from Gore et al. (2020) and Zeibell and Roberston (2021) document negative effects on teacher workload and wellbeing. Despite spikes in anxiety just prior to school closures and again prior to schools reopening, one rare exception is a survey of teachers in the UK which found improved wellbeing among teachers during school lockdowns as “the day-to-day stress of managing classrooms was removed” (Allen et al., 2020, p. 18).

We see this paper as our contribution to building a much-needed comprehensive picture of the effects of COVID on teachers—foundational to providing adequate support to an “already weary profession” (Dabrowski, 2020, p. 37) and ensuring predicted teacher shortages (Henebery, 2020) are not exacerbated. We focus on the experiences of teachers in NSW, Australia, both as they rapidly moved to learning from home *and* when they returned to face-to-face teaching in 2020. Because we were in the middle of conducting a randomised controlled trial (RCT) when COVID struck, we were uniquely positioned to compare quantitative indicators of self-efficacy, morale, and sense of appraisal and recognition for a cohort of teachers affected by the pandemic (data collected in 2020) with a cohort of teachers unaffected by the pandemic (data collected in 2019).

While efficacy has been widely studied and theorised (Goddard et al., 2004; Klassen et al., 2011; Tschannen-Moran et al., 1998), empirical research on teacher morale and sense of appraisal and recognition is less extensive (Evans, 1997; Mackenzie, 2007; Whiteoak, 2020). We use quantitative indicators (self-efficacy, morale and a sense of appraisal and recognition) combined with teachers’ own accounts of the effects of the pandemic on their working lives, to explore how teachers were impacted by the pandemic. Morale and efficacy were not directly addressed in the interviews but came to the fore in our deductive analysis.

## Methodology

### Research context

We did not set out to study the effects of COVID on teachers. Our mixed-methods randomised controlled trial (RCT) examined the effects of Quality Teaching Rounds professional development, split across cohorts in 2019 and 2020, on student achievement, the quality of teaching, teacher morale, efficacy, appraisal and recognition. We were nearing the completion of baseline data collection in 2020 for the second cohort when COVID struck. We had pre- and post-intervention data from 2019 and pre-intervention data in 2020 for most schools in the second cohort before the late March closure of schools in NSW. In short, these data enabled the comparison of outcomes from 2020 with those from a cohort of teachers and students in 2019 who had not been disrupted by the pandemic.

Given the relatively low number of COVID cases in Australia in 2020, schools in NSW reopened with ample time to collect follow-up data at the same time of year as the 2019 post-intervention data collection—commencing in late October and concluding in early December. Just as the global crisis was worsening and schools were still shut down or shutting down in many parts of the world, the situation in NSW enabled us to go back into schools to investigate effects on teachers and on student learning once they returned to the classroom, a unique vantage point in the research conducted to date (see Gore et al., 2021 for findings regarding student learning).

The government schooling system in NSW only implemented learning from home (delivering lessons remotely and providing on-site care for children of essential workers) for 8 weeks prior to reopening in Term 3, 2020 for all students. Once schools reopened, however, extensive restrictions to usual school practices were mandated (NSW Department of Education, 2020), such as the cancellation of school excursions, assemblies, sporting activities and large gatherings (Australian Government Department of Health, 2020).

The geographic isolation of Australia, strict hotel quarantine processes for returned travellers and sharp lockdowns to stem potential outbreaks were relatively effective in containing the virus until the middle of 2021. As such, the situation in NSW has been one of relative wellbeing, compared to other nations. Nonetheless, the documented experiences of teachers in our study signal a warning for teachers and students who face longer periods away from their classrooms.

### Data sources and analytical methods

In the following section, we provide details of the survey, the interview schedule and the analytical methods used for this paper.

#### Teacher surveys

As part of our *Building Capacity for Quality Teaching in Australian Schools* project Miller et al., 2019, 2021), surveys were conducted with one cohort of Year 3 and Year 4 teachers in 2019 and another cohort of Year 3 and Year 4 teachers in 2020. The survey, administered at multiple time points during 2019 (Terms 1, 2, 3 and 4) and 2020 (Terms 1, 3 and 4), included scales for teacher efficacy (Tschannen-Moran & Hoy, 2001), collective morale and sense of appraisal and recognition (Hart et al., 2000). The efficacy scale includes sub-scales for student engagement, instructional strategies and classroom management. The morale scale focuses on teachers’ perceptions of morale among their school colleagues. The appraisal and recognition scale addresses the provision of feedback and recognition of staff performance. The collection of baseline data in 2020 and control group data in 2019 enabled us to provide insight into the impact of COVID on these variables.

School and teacher demographics for the sample are presented in Table 1. As depicted, schools that participated in the 2019 study as part of the control group were similar to those in the 2020 cohort in terms of geographic spread, teachers’ average years of teaching experience and teaching qualifications (Table 1). Some relatively minor differences were apparent, however. Compared to the schools in the 2019 cohort, schools in the 2020 cohort were more likely to be located in major cities (78% in 2020 compared to 56% in 2019) and less likely to have ICSEA scores less than 950 (20% in 2020 compared to 31% in 2019), and teachers in the 2020 cohort had slightly less teaching experience on average than the teachers in the 2019 cohort (10.1 years in 2020 compared to 11.8 years in 2019) (Table 1).

**Table 1:** Characteristics of teachers and their schools in survey sample (2019, 2020)

Characteristics	2019	2020
Schools, *n*	62	51
ICSEA, mean (SD)	995 (82)	1007 (76)
ICSEA < 950,* n* (%)	19 (31)	10 (20)
ICSEA 950–1049,* n* (%)	29 (47)	25 (49)
ICSEA 1050 + ,* n* (%)	14 (23)	16 (31)
Rural,* n* (%)	27 (44)	11 (22)
Major cities	35 (56)	40 (78)
Inner regional	21 (34)	10 (20)
Outer regional	5 (8)	1 (2)
Remote	0 (0)	0 (0)
Very remote	1 (2)	0 (0)
Teachers, *n*	228	119
Experience—years, mean (SD)	11.8 (9.3)*	10.1 (8.3)
Qualifications—Masters (%)	30 (15)	16 (13)
Qualifications—Bachelor (%)	151 (77)	94 (79)
Qualifications—Diploma (%)	16 (8)	6 (5)

*ICSEA* index of socio-educational advantage, *SD* standard deviation

*Obtained from 197 (86% of total) completed demographic surveys

Survey data were analysed using IBM PASW Statistics 27 software (SPSS Inc. Chicago, IL). Linear mixed models were fitted to compare the teacher perception outcomes for each of the cohorts (2019 and 2020) at each of the measured time points. Categorical variables of year (2019 and 2020), time (Term 1, Term 3 and Term 4) and the interaction of year-by-time were assessed as fixed effects within the models (the Term 1 results for the 2019 cohort were the reference category in all models). As all participants in the 2020 cohort had missing values at the Term 2 time point, this time point was excluded from analysis for both cohorts. To account for the correlation among repeated measures within individuals and clustering of individuals within schools, a repeated statement specifying an unstructured covariance pattern and a random intercept modelling the variance components at the school level were included in the model. In some models, there was not enough variance attributable to the school level (indicated by a non-significant variance parameter) and the random intercept was removed. Significance levels (< 0.05, < 0.01 and < 0.001) are presented for the reader. No adjustments for multiple outcome assessment were made to the results of significance tests.

To evaluate the effect of missing data on the outcomes, results of the available (‘original’) data were compared for those who completed the survey at all three time points (2019 = 98/228; 2020 = 63/119) and for a complete imputed data set. As the missing data patterns were non-monotone and these data did not demonstrate multivariate normality (univariate skewness ranged from − 0.1 to − 0.9), data were imputed using fully conditional specification in SPSS. This procedure uses multivariate imputation by chained equations (MICE) which does not rely on the assumption of multivariate normality (Fitzmaurice et al., 2011). The imputation included the outcome variables, year (2019, 2020) and time (Term 1, Term 3, Term 4) as covariates. Observed covariates of teaching experience (continuous) and school ICSEA (continuous) were included in addition to the variables included in the planned analysis to improve precision and reduce bias in line with increasing plausibility of the assumption of data being missing at random. Two-way interactions were included for categorical variables (year and time). Twenty imputations were obtained using 500 iterations.

#### Teacher interviews

To gain a deeper understanding of the nature and effects of the pandemic on teachers, teachers and school leaders from a representative sample of schools were invited to take part in semi-structured telephone interviews. While interviews with teachers provided an in-depth understanding of how individual teachers fared during the school closure period, interviews with school leaders provided a means of gauging how widespread issues were, given their broader school-wide perspective.

Interviews were conducted during September and October 2020 with teachers (*n* = 12) and school leaders (*n* = 6) from 13 primary schools drawn from the broader study and representing a broad cross section of NSW schools. The sample demographics of the schools involved are displayed in Tables 2 and 3. There were slightly more schools in regional areas (*n* = 7; inner regional,* n* = 4; outer regional,* n* = 3) than in major cities (*n* = 6). The ICSEA of these schools ranged from just over 800 (least advantaged school) to around 1140 (most advantaged). The percentage of students in these schools with a language background other than English (LBOTE) ranged from 0% (Schools 2 and 4) to more than 95% (School 5). Aboriginal and Torres Strait Islander student enrolment ranged from 1% in School 5 to around 60% in School 1. The number of classroom teachers ranged from 3 (School 4) to 41 (School 13), and student enrolments ranged from just under 30 (School 4) to around 750 (School 13) (Table 3).

**Table 2:** Location of 2020 interview schools and participants

	Major city	Inner regional	Outer regional	Total
Schools	6	4	3	13
School leaders	3	2	1	6
Teachers	5	4	3	12

**Table 3:** Sociodemographic characteristics of schools in interview sample

	ICSEA	Language background (%)	Aboriginal or Torres Strait Islander students (%)	Number of teachers (2019)	2019 enrolment
School 1	Low	< 10	61–70	31–40	451–500
School 2	Low	< 10	31–40	< 10	51–100
School 3	Low	< 10	41–50	11–20	251–300
School 4	Low	< 10	< 10	< 10	< 50
School 5	Low	91–100	< 10	21–30	301–350
School 6	Mid	11–20	11–20	21–30	451–500
School 7	Mid	< 10	< 10	11–20	251–300
School 8	Mid	< 10	< 10	< 10	151–200
School 9	Mid	71–80	< 10	11–20	251–300
School 10	Mid	< 10	< 10	11–20	301–350
School 11	Mid	< 10	< 10	21–30	351–400
School 12	Mid	11–20	< 10	11–20	251–300
School 13	High	71–80	< 10	41–50	751–800

In order to protect the anonymity of schools, ICSEA is reported as low (< 950), mid (ICSEA 9501–1050), and high (ICSEA > 1050) and all other variables are reported as a range

Following verbatim transcription, each interview was entered into NVivo 12 (QSR International, 2020), a qualitative software analysis tool used to assist in thematic coding of interviews. The coding process, using deductive logic (Creswell, 2013), identified key themes from participants’ responses to questions about the learning from home period, including ‘What effect (if any) has the break from traditional schooling had on staff well-being?’ (see Appendix 2 for a full list of interview questions). Following each stage of coding, two researchers met to discuss and compare their codes to ensure consistency and expand or combine themes as required (Harry et al., 2005). The first wave of this process generated 26 codes while discussion of codes at a meeting of all researchers reduced the number of codes to 22. This refined set of codes guided the remaining coding of interviews. The final list of codes was grouped into the following categories: advantages of learning from home, challenges to learning from home; experiences of learning from home, impacts of learning from home, learning from home arrangements, support for learning from home and return to school experiences. Finally, researchers met to discuss codes and key themes. In this paper, we draw on those codes relating specifically to the impact of COVID on teachers. When reporting interview data in this paper, our references to ‘most teachers’ or ‘some teachers’ indicate the frequency of responses among our participant cohort; we make no claims to represent the views of all teachers in NSW. In reporting the substance of the interviews, pseudonyms are used to ensure the confidentiality of participants and schools involved in the study.

## Results

### Teacher surveys

**Fig. 1:**
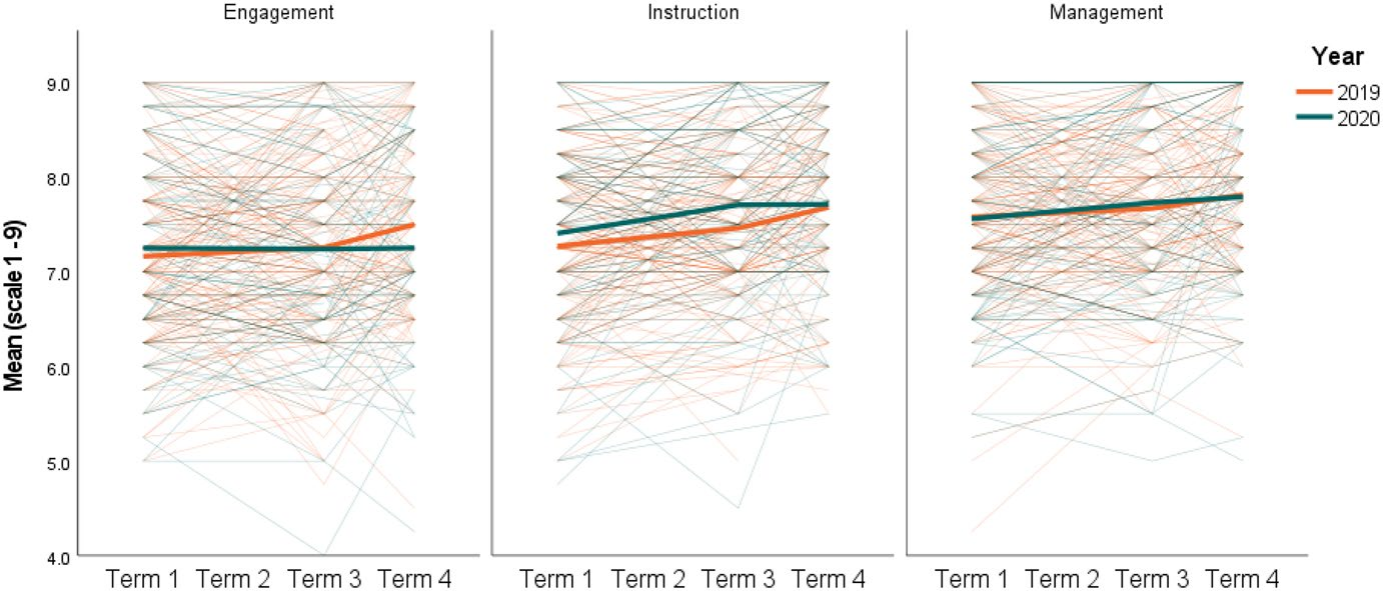
Teaching efficacy—original data. (Color figure online)

**Fig. 2:**
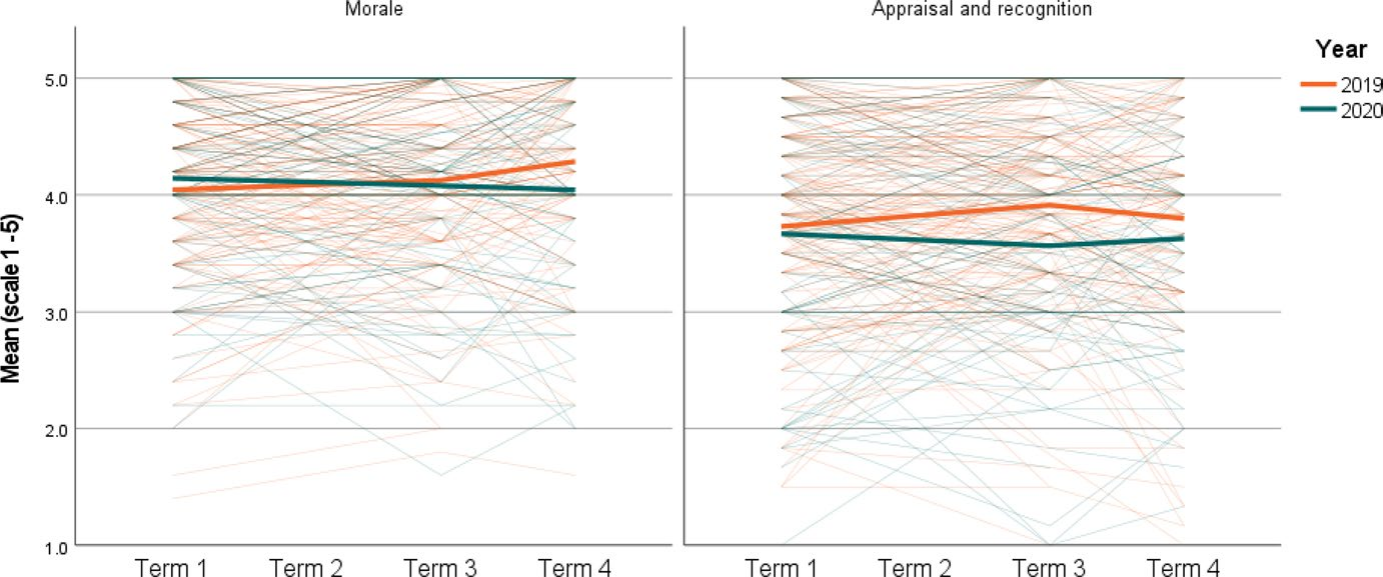
Teacher morale and appraisal and recognition—original data. (Color figure online)

Results of the teacher survey analysis are displayed in Tables 4, 5, 6 and 7 and depicted visually in Figs. 1 and 2. Table 4 displays the mean for each outcome and the response rate in each school term. Response rates in relation to the number of participants at baseline were similar for both cohorts at the Term 3 timepoint (~ 62% of baseline), however a greater proportion of the 2020 cohort (66%) completed the final survey (Term 4) than the 2019 cohort (53%). This was likely due to the special request to complete the survey and the lack of other data collection at the time due to cancellation of the RCT. Overall, response rates were high.

**Table 4:** Teacher survey sampling and descriptive statistics

Outcome	Time (T)	2019		2020	
		Mean (SD)	*N* (% of T1)	Mean (SD)	*N* (% of T1)
Engagement	T1	7.16 (0.92)	228	7.25 (1.04)	119
	T3	7.26 (0.98)	145 (63)	7.23 (1.09)	74 (62)
	T4	7.50 (0.91)	123 (53)	7.25 (1.05)	78 (66)
Instruction	T1	7.27 (0.91)	228	7.41 (0.98)	119
	T3	7.46 (0.83)	145 (63)	7.71 (0.95)	74 (62)
	T4	7.68 (0.87)	123 (53)	7.69 (0.90)	78 (66)
Management	T1	7.58 (0.89)	228	7.56 (0.97)	119
	T3	7.67 (0.84)	145 (63)	7.72 (1.03)	74 (62)
	T4	7.82 (0.80)	123 (53)	7.77 (1.00)	78 (66)
Morale	T1	4.04 (0.85)	228	4.14 (0.75)	119
	T3	4.13 (0.75)	145 (63)	4.08 (0.81)	73 (61)
	T4	4.29 (0.74)	123 (53)	4.04 (0.87)	78 (66)
Appraisal and recognition	T1	3.73 (0.92)	228	3.67 (1.02)	119
	T3	3.91 (0.84)	145 (63)	3.58 (1.03)	73 (61)
	T4	3.80 (0.95)	123 (53)	3.63 (0.96)	78 (66)

*T* school term, *SD* standard deviation, *N* total sample population

**Table 5:** Baseline characteristics (completers vs non-completers)

	Group	Complete		Non-complete		Difference			
		Mean (SD)	*N*	Mean (SD)	*N*	Mean	Effect (d)	*t* (df)	*p*
Engagement	2019	7.10 (0.90)	98	7.21 (0.93)	130	0.11	0.12	0.821 (226)	0.412
	2020	7.26 (1.09)	63	7.24 (1.00)	56	− 0.02	− 0.02	− 0.088 (117)	0.930
Instruction	2019	7.29 (0.82)	98	7.26 (0.97)	130	− 0.03	− 0.03	− 0.266 (226)	0.790
	2020	7.42 (0.98)	63	7.39 (0.98)	56	− 0.03	− 0.03	− 0.179 (117)	0.858
Management	2019	7.52 (0.84)	98	7.62 (0.92)	130	0.10	0.11	0.829 (226)	0.408
	2020	7.51 (1.02)	63	7.61 (0.91)	56	0.10	0.10	0.558 (117)	0.578
Morale	2019	4.11 (0.75)	98	3.99 (0.91)	130	− 0.12	− 0.14	− 1.060 (226)	0.290
	2020	4.12 (0.75)	63	4.16 (0.76)	56	0.04	0.05	0.292 (117)	0.771
Appraisal and recognition	2019	3.84 (0.84)	98	3.65 (0.97)	130	− 0.19	− 0.21	− 1.551 (226)	0.122
	2020	3.66 (1.01)	63	3.67 (1.04)	56	0.01	0.01	0.060 (117)	0.953
Experience	2019	13.21 (9.85)	98	10.74 (8.73)	130	− 2.47	− 0.27	− 2.005 (226)	0.046
	2020	10.05 (8.15)	63	10.18 (8.58)	56	0.13	0.02	0.085 (117)	0.932
ICSEA	2019	1013 (78.37)	98	988 (81.79)	130	− 25	− 0.31	− 2.402 (226)	0.017
	2020	1016 (78.26)	63	1012 (76.52)	56	− 4	− 0.05	− 0.274 (117)	0.784

**Table 6:** Teacher efficacy, teacher morale and appraisal (2019–2020)

	Teacher efficacy			Morale	Appraisal and recognition
	Engagement	Instruction	Management		
Fixed effects					
Initial status					
Intercept (95% CI)	7.16 (7.04 to 7.29)*	7.27 (7.15 to 7.39)*	7.58 (7.46 to 7.69)*	4.05 (3.91 to 4.20)*	3.74 (3.58 to 3.90)*
Baseline difference (95% CI)	0.09 (− 0.13 to 0.30)	0.14 (− 0.07 to 0.34)	− 0.02 (− 0.22 to 0.18)	0.08 (− 0.15 to 0.31)	− 0.09 (− 0.34 to 0.16)
Change over time—2019 cohort					
Term 1–Term 3 (95% CI)	0.09 (− 0.06 to 0.24)	0.14 (0.02 to 0.27)*	0.07 (− 0.07 to 0.19)	0.06 (− 0.05 to 0.16)	0.13 (0.01 to 0.26)*
Term 1–Term 4 (95% CI)	0.35 (0.20 to 0.49)*	0.41 (0.28 to 0.54)*	0.23 (0.09 to 0.36)*	0.16 (0.06 to 0.26)*	− 0.01 (− 0.15 to 0.13)
Change over time to 2020 vs 2019					
Term 1–Term 3 (95% CI)	− 0.13 (− 0.39 to 0.12)	0.12 (− 0.10 to 0.34)	0.08 (− 0.15 to 0.30)	− 0.13 (− 0.31 to 0.05)	− 0.19 (− 0.41 to 0.02)
Term 1–Term 4 (95% CI)	− 0.28 (− 0.52 to − 0.04)*	− 0.05 (− 0.27 to 0.16)	0.06 (− 0.17 to 0.28)	− 0.24 (− 0.41 to − 0.08)*	− 0.04 (− 0.27 to 0.19)
Effect size over time to 2020 vs 2019					
Term 1–Term 3 (95% CI)	− 0.14 (− 0.39 to 0.12)	0.13 (− 0.11 to 0.38)	0.09 (− 0.17 to 0.34)	− 0.16 (− 0.38 to 0.06)	− 0.21 (− 0.43 to 0.02)
Term 1–Term 4 (95% CI)	− 0.29 (− 0.53 to − 0.04)*	− 0.06 (− 0.30 to 0.18)	0.06 (− 0.19 to 0.31)	− 0.30 (− 0.51 to − 0.10)*	− 0.04 (− 0.28 to 0.20)
Variance components					
Intercept (School)	N/A	N/A	N/A	0.21 (0.14 to 0.31)*	0.18 (0.1 to 0.32)*
Change over time to 2020 vs 2019					
Completers					
Term 1–Term 3 (95% CI)	− 0.23 (− 0.52 to 0.06)	0.11 (− 0.14 to 0.35)	0.05 (− 0.21 to 0.3)	− 0.07 (− 0.27 to 0.12)	− 0.15 (− 0.40 to 0.09)
Term 1–Term 4 (95% CI)	− 0.30 (− 0.59 to − 0.01)*	− 0.07 (− 0.33 to 0.19)	0.07 (− 0.2 to 0.34)	− 0.19 (− 0.38 to 0.00)*	− 0.02 (− 0.28 to 0.25)
Imputed data					
Term 1–Term 3 (95% CI)	− 0.10 (− 0.40 to 0.20)	0.12 (− 0.14 to 0.39)	0.11 (− 0.15 to 0.37)	− 0.12 (− 0.37 to 0.12)	− 0.27 (− 0.56 to 0.01)
Term 1–Term 4 (95% CI)	− 0.26 (− 0.57 to 0.05)	− 0.05 (− 0.32 to 0.22)	0.06 (− 0.22 to 0.34)	− 0.32 (− 0.56 to − 0.07)*	− 0.10 (− 0.40 to 0.21)

*CI* confidence interval

*p < 0.05; **p < 0.01; ***p < 0.001

Table 5 outlines the differences for individuals with complete and missing data, respectively. While not statistically significant, effect sizes greater than *d* $\pm$ 0.10 were observed between completers and non-completers for the outcomes of engagement, morale and appraisal in the 2019 cohort. Experience levels and school ICSEA were also significantly lower for those who did not complete all surveys in 2019. Given that the available data appeared to have some relation to the missingness, analysis proceeded under the assumption that data were missing at random.

Appendix 1 presents the mean result for each group at each timepoint for the original, complete and imputed data. The imputed data do not display any consistent pattern in relation to the other groups of data with no sign of extreme values being created by the imputation process. The statistical analysis of the trends among outcomes for both cohorts is presented in Table 6.

Analysis of the original data demonstrated significant negative effects for teacher efficacy in relation to student engagement and for teacher morale. The 2019 cohort displayed a small, non-significant increase in engagement and morale between Term 1 and Term 3 (Change over time—2019 cohort), with a significant lift in both outcomes to the final time point (Term 4). By comparison (Change over time—2020 vs 2019), teachers in the 2020 cohort displayed almost identical results for engagement efficacy between Term 1 and Term 3 and a marginal drop in their engagement efficacy to be significantly below the 2019 cohort at the Term 4 time point (− 0.28; 95% CI   − 0.52 to − 0.04; *p* < 0.05). Similarly, for the morale outcome, the 2020 cohort displayed a small but consistent downward trend across the year and was significantly lower than the 2019 cohort at the Term 4 time point (− 0.24; 95% CI   − 0.41 to − 0.08; *p* < 0.05).

Expressed as an effect size, the differences between 2019 and 2020 cohorts for both the engagement efficacy and morale outcomes were approximately *d* =  − 0.15 at Term 3 and *d* =  − 0.30 at Term 4. While these differences were statistically significant, the effect sizes are considered small. When considering the scales associated with the questions banks (Efficacy = 1 to 9 and Morale = 1 to 5), the differences between the groups at the final time point represent a mean response of 7.51 for the 2019 cohort and 7.25 for the 2020 cohort for engagement efficacy and 4.29 versus 4.07 for the average morale response among 2019 and 2020 cohorts, respectively. These differences may or may not represent a practically significant impact on the efficacy and morale of the 2020 cohort, but they suggest a negative trend. No significant effects were found for teacher efficacy in relation to instructional strategies or classroom management, or for appraisal and recognition.

In terms of evaluating bias within the data due to missingness, the significance values for both engagement and morale changed minimally between the original, complete and imputed data (likely an effect of the different sample sizes). However, the small amount of variability between the year-by-time parameter estimates for the different data sets provides confidence that there is an underlying effect among the 2020 cohort for these outcomes.

Figures 1 and 2 provide a graphical illustration of the trends and highlight the enormous variability among individual teachers, evident in the finer lines depicting change over time for each participant. Despite this variability, overall there is evidence of a downward trend for morale and engagement efficacy in 2020 relative to 2019.

### Teacher interviews

In the following section, we analyse the interview data to better understand how teachers and school leaders were impacted during and after the learning from home period. When provided with an opportunity to discuss their experiences during the first wave of the pandemic, two overarching themes emerged despite no direct questioning about these matters: flagging morale and declining self-efficacy. Representative extracts are used to highlight these key themes and serve to illuminate the small but significant findings from the quantitative analysis.

#### Flagging morale

During the remote learning period, teachers delivered lessons in a variety of modes—through online programs of work, by creating paper-based learning resources for students with limited access to technology, as well as classroom supervision for children of essential workers. Although schools differed in their use of technology and resources, school leader Kylie’s description of lesson delivery during the closedown period captures the intensive experience of many of the participating teachers during this time:

We made sure that every child had access to some learning, so we hand-delivered paper packs to families who weren’t engaging online. The teachers created a weekly, and then daily, schedule of suggested outcomes, suggested learning, and that was posted online or delivered in the paper packs. We sent lots of letters home to parents just saying, you know, “Do what you can, but make sure, or try and make sure, your child does some online learning or some paper learning”. We gave out stationery, exercise books, readers, some sport equipment, and basically, we wanted the parents to show the teacher that they’d done some work. (Kylie, school leader, school 9, major city, mid ICSEA)

Prior to COVID, the intensification of work (Williamson & Myhill, 2008), deteriorating morale (Mackenzie, 2007; Stroud, 2018; Whiteoak, 2020) and the rise of performativity (Ball, 2003; Sullivan et al., 2020) were already affecting the teaching workforce in NSW. The intensification of labour described by Kylie contrasts sharply with the usual classroom-based practice of most primary school teachers, and clearly demonstrates how COVID not only amplified workplace issues but added a new layer of pressure. Teachers reinvented lesson plans to allow for different forms of delivery and pivoted to new ways of working with a constant eye on how to keep their students engaged. As one classroom teacher put it, “the workload was really overwhelming, and I felt like we had to reinvent the wheel each day” (Chris, teacher, school 11, major city, mid ICSEA).

Many teachers also reported struggling in their dual roles as parents and teachers. With the combination of increased school workloads and their own caring responsibilities, they felt apprehensive and undervalued. One school leader explained, “There were some [teachers who] were highly, highly anxious; there were ones who were juggling elderly parents…their own kids, in different schools” (Rachel, school leader, school 13, major city, high ICSEA). Another recounted that, overall, staff were “demoralised and not valued”, “They just felt they didn’t count” and would “walk away” from the profession if they could (Lauren, school leader, school 6, major city, mid ICSEA).

Contradictions in government policy that advocated social distancing and working from home while simultaneously asking students (and therefore teachers) to return to school led to teachers feeling vulnerable and confused. Classroom teacher Daniel, for example, said:

I didn’t understand how it was that social distancing had to be observed, hygiene had to be observed, everybody had to self-isolate and work from home if they could, and yet you were going to put me into a room with 30 kids! That worried me. … I suppose it was frightening to know every other workplace had been told that they can’t sit next to each other and “work from home if you can”, and yet I just had a kid sneeze in my eye. And that’s okay because you’re telling me that I can’t catch it off a kid? I found the mixed messages there – telling society one thing and teachers another – that was quite hard to deal with. (Daniel, teacher, school 6, major city, mid ICSEA)

This mixed messaging during the height of the pandemic in 2020 increased teachers’ feelings of unease. Daniel’s use of the term *frightening* conveys a deep concern about contracting the virus in the workplace.

In many schools, flagging morale was exacerbated by perceptions of poor communication and lack of support from the government and the Education Department.

I think the Department [was] caught between a rock and a hard place. As a principal, I didn’t feel particularly well supported. We were doing extraordinary hours, and it was … you know, the changing landscape, and the time they were communicating with us as principals, at 11 or 12 o’clock at night. …They felt *they’d* ticked the box by getting it out late at night, but that doesn’t mean you can have that up and running for the next day at school, because there’s turnaround [time needed] in communication. (Rachel, school leader, school 13, major city, high ICSEA)

As an experienced principal, Rachel acknowledges the constraints on the Department, *caught between a rock and a hard place.* She also vividly captures the extraordinary impact on school leaders. Receiving imperatives to send and receive messages late at night was viewed as impossible to implement.

For teachers in rural communities, the communication challenges were even more fraught. Poor infrastructure, limited access to 4G networks and to quality teaching resources from the Department left some teachers feeling undervalued and adrift. Teachers, like Andrew from an outer regional school, felt frustrated by not providing students with the same quality learning experiences received by children in major cities:

I think the Department really worked on thanking us more than anything, and we didn’t need thank you because we were doing our jobs. What we needed was that support, [to be told] “what you’re doing is okay”. I don’t think they really got that message out. It was more like a “this is what we have to do, this is the benchmark”, sharing all the top things that teachers were doing. But those teachers have access to 4G networks and, you know, social hubs within urban areas. And we couldn’t really match that at all. So, very quickly myself and the principal saw inequalities in what we were delivering to our kids, very quickly. And that crushes the spirit when you’re truly really trying to give, like provide a quality education. So yeah, I saw a really big imbalance about what our kids were going to receive out here as opposed to kids in urban areas. …That’s where I feel like that we weren’t supported. (Andrew, teacher, school 4, regional, low ICSEA)

Clearly Andrew is more concerned about equity for his students than gratitude for his efforts. He and his principal worried about being unable to *match* the delivery of online teaching that they perceived to be occurring in metropolitan schools to such an extent that it *crushes the spirit*. As an educator committed to his students’ learning, he reports being acutely aware of disparities that he felt were not acknowledged by the system.

Most interestingly, life did not necessarily improve for teachers once students returned to school. As Lauren, a school leader in an urban location, points out, the increased workload was unrelenting and did not dissipate at the end of the 8-week learning from home period:

Double the workload. I think I’ve seen that in nearly everyone. Double the workload. Teachers now feel like now we’ve come back to school, they now feel like they have to catch up on all the content that they missed due to our overcrowded syllabus... So, teachers are now very stressed that they have to catch up on this syllabus. Teachers, myself, my DP [Deputy Principal], we’re all struggling with the behaviour of students, and this is affecting teachers’ wellbeing hugely, absolutely hugely. We're in-school suspending, we're evacuating classrooms. The behaviour has really ratcheted up a notch. (Lauren, school leader, school 6, major city, mid ICSEA)

Lauren’s sharp commentary conveys the stressful context of return. Repetition of *double the workload* and pressure to *catch up* shows already exhausted teachers trying to cope. The bleak picture is further exacerbated by troubling student behaviour leading to classroom evacuation and suspension from school.^1^

While the survey analysis revealed a significant negative impact on teacher morale, these interviews with teachers and school leaders exposed a multitude of factors that adversely influenced their morale during 2020—the intensification of labour, a perceived lack of support during learning from home and challenges once teachers returned to face-to-face teaching.

#### Declining teacher self-efficacy

It is widely documented that teachers with greater self-efficacy are more resilient when faced with challenges than colleagues with lower self-efficacy (Tschannen-Moran et al., 1998). Nevertheless, during times of crisis, we might expect teachers to experience role overload (Kuntz et al., 2013), as illustrated above, leading to lower self-efficacy (Seyle et al., 2013) and reduced sense of success (Kraft et al., 2020). While our statistical analysis found little change between 2019 and 2020 in teachers’ efficacy in relation to classroom management and instruction, there was a significant difference in how well they felt able to engage their students. The interviews provide a more vivid picture, with teachers expressing feelings of inadequacy, frustration and limited ability to engage their students—both during learning from home and upon return to school.

For example, Andrew, a classroom teacher from an outer regional low ICSEA school, reported feelings of inadequacy as he grappled with the rapid move to learning from home:

There were times when I felt, I did feel inadequate. There were times where I thought “I can't help these kids”… There were times I thought “oh, I’m really not doing my job well”, you know “you should be really prepared for any type of learning”, and I didn’t really feel like that at all. (Andrew, teacher, school 4, regional, low ICSEA)

Andrew wrestled with his sense of efficacy throughout the learning from home period as the sudden shift to a new way of teaching left him feeling ill-prepared. Other teachers, such as Chris, a teacher from a mid ICSEA school in a major city, articulated not only his exhaustion but ongoing frustration at feeling powerless to deliver content in ways that were satisfying and educationally sound.

I think we all share the same frustrations. We were all exhausted. …as well as feeling like you’re not providing good content and then the students aren’t learning the way they should be. And then also not being able to teach in the way that you feel best. I think those were all shared frustrations between the whole group. (Chris, teacher, school 11, major city, mid ICSEA)

Despite flagging morale (previous section), exhaustion and perceived lack of support, teachers were committed to the education of their students. But the frustrations of working under entirely unfamiliar conditions led some teachers, like Chris (above), to question their efforts. Here, Daniel, a classroom teacher from an inner regional area, offers a grim description of a staff meeting to discuss teachers’ widespread concerns with student engagement:

We kind of all felt as though it wouldn’t really matter how much effort we put in on our side, or how much time or money was spent on resources, or whatever it may be, because ultimately the engagement really wasn’t there from both kids and their families. And so, it was sort of like, is it worth breaking our necks to try and do more, or do we ride this out for a couple of weeks longer? Because it’s not, possibly, going to make a difference. (Daniel, teacher, school 6, major city, mid ICSEA)

This is a bleak picture indeed. Rapidly declining student engagement and lagging self-efficacy in teachers led to recognition that teaching *doesn’t* *really matter* and *won’t* *make a difference* to student achievement. And this is after only an 8-week period of learning from home.

Particularly worrying is that teachers saw little improvement once students returned to the classroom. Poor student engagement in classroom activities continued to challenge teacher self-efficacy, as Mateo, a classroom teacher from an urban area illustrates:

And even the engagement, their concentration levels really, really dropped off a lot. Focusing... they can’t sit still for more than a minute and like I said, normally before COVID, they were fine. They were able to participate in class discussions. And all of a sudden now, engagement... they can’t sit still anymore. They’ve always got to be up. Focus and concentration floats in and out… routine is gone, it's not there anymore. (Mateo, teacher, school 13, major city, high ICSEA)

Mateo paints a vivid picture of fractured classrooms with disengaged students and a lack of routine. The ability to focus, to sit still and concentrate are preconditions for learning, but students (like their teachers) appeared very tired, ‘not engaging as much, lots of behaviour issues’ (Samantha, teacher, school 1, outer regional, low ICSEA). While there is limited research examining student engagement in learning during COVID (Borup et al., 2020; Khlaif et al., 2021), our evidence suggests that disengagement not only continued after students returned to face-to-face schooling, but had an ongoing impact on teacher self-efficacy and their power to deliver quality teaching.

In summary, many of the teachers we interviewed were unable to teach in a way they felt was appropriate for their students and, despite their best efforts, felt they were unable to have a positive impact on student engagement, including during the period when students returned to school. Declining teacher self-efficacy, underpinned by feelings of inadequacy, frustration, exhaustion and poor student engagement impacted significantly on teachers during COVID. Rekindling teacher self-efficacy will require positive support for teachers and recognition of the remarkable role they played in educating students during the pandemic.

#### Exceptions

Not every teacher in our study was impacted negatively by the challenges of the pandemic. There is some evidence that unexpected change, such as that brought about during crisis situations, can also have positive effects (Haski-Leventhal, 2020). As signalled by the variation in responses to our teacher survey (Figs. 1, 2), and despite the changes to teaching imposed by the pandemic, some school leaders reported positive outcomes and growth in teachers’ confidence as a result of having to transform their teaching practice on short notice. School leader Katherine praised the resilience and ability of her teachers to engage with new technologies and develop new forms of pedagogy capably and efficiently.

We learnt that we could do many things, very difficult things, very quickly. Some of my staff who were not tech [savvy]… got really, not afraid of Zoom, [but] they had to learn something that was very foreign to them, and many of them were very petrified, but they did it, and they’re not afraid of that anymore. So that’s a big bonus. (Katherine, school leader, school 12, inner regional, mid ICSEA)

A second example comes from school leader James who detailed how the learning from home period improved the quality of pedagogical practice:

Probably the *only* positive that we can pinpoint as a staff …is … it forced us to be adaptable. It forced us to not just sit with what we had traditionally done and say we’re always going to do that. So, it was the force of change if that makes sense and we found that a positive in that we were really questioning our delivery of our teaching… So, we went “okay, we’ve actually got to think about our delivery of our teaching and learning to ensure that what we want to be learnt is being learnt”. (James, school leader, school 4, outer regional, low ICSEA)

Clearly teachers had to move away from traditional forms of teaching during the shutdown period, yet for some it led to greater introspection and adaptability, and new insights into their practice. James is quick to point out, however, that this was the *only* positive benefit.

Overall, the qualitative data not only confirm the patterns from the statistical analysis of reduced morale and self-efficacy (engagement) for the teachers affected by COVID during 2020 relative to the 2019 cohort. The interviews also fortify these effects, with the themes of morale and student engagement paramount in teachers’ accounts of how COVID impacted on them and their teaching.

## Discussion

Globally, more than 1.5 billion school students and their teachers were affected in 2020 by school closures in response to the COVID pandemic (UNESCO, 2020), the ongoing effects of which are still largely unknown. This study provides timely evidence on the impact of COVID on teachers, a relatively neglected area of study. Our quantitative data revealed significant negative effects for both teacher efficacy in relation to student engagement and teacher morale. Teachers in 2020 reported lower levels of morale and felt less able to engage their students in learning than teachers in 2019, while no significant effects were found for teacher efficacy in relation to instructional strategies or classroom management, or for appraisal and recognition.

Some limitations should be considered when interpreting the quantitative results of this paper. First, the broader study in which teachers were involved focussed on effects at the student level, with teacher characteristics and outcomes not accounted for within randomisation, meaning cohort-based characteristics could have impacted the results of this study. However, there were no significant differences between the two cohorts at baseline (Term 1) for any of the measured variables (see Table 7—Appendix 1), indicating that cohort influences are likely to be minimal. Second, the relatively large amount of missing data may have had an impact on the findings from these data. However, estimates from all three data sets (original, completers and imputed) demonstrate the relative consistency of the model estimates. Finally, while the data were originally gathered for a different study, they provide rigorous comparable evidence obtained just prior to the pandemic, enabling a greater understanding of the impact of school closures on teachers.

Similar to previous research, this study found significant negative impacts on teachers as a result of changes to schooling associated with the pandemic (Alves et al., 2021; Hascher et al., 2021; Zeibell & Robertson, 2021). Furthermore, going beyond studies examining the impact of longer-term school closures (Allen et al., 2020; Kraft et al., 2020), our study demonstrates that even relatively short-term closures can have significant negative impact on teachers.

Importantly, the period of school closure and challenges associated with the return to school seriously impacted teacher morale and challenged their capacity to engage students (Kraft et al., 2021; Kurtz et al., 2020). Shaken by substantial changes to familiar approaches to teaching and learning, our data demonstrate that teacher morale and efficacy are subject to change and were affected by a range of factors during this period of crisis. Moreover, in line with the previous research on teacher morale during COVID-19, our results indicate that teacher morale and efficacy are in need of nurturing especially when changes to schooling challenge their wellbeing and sense of themselves as teachers (Kurtz et al., 2020).

Notably, all 18 teachers interviewed for this study reported negative effects related to COVID with few reporting any positive effects. Unlike research undertaken with schools in the United States which found greater impact on teachers in disadvantaged schools (Kraft et al., 2021), in our study the impact on teachers did not appear to vary by school ICSEA. On the other hand, we did find that teachers in regional and remote areas often faced significant additional burdens, most notably related to differential access to reliable internet (Halsey, 2018; Masters et al., 2020). The measurable decrease in teachers’ morale during 2020 could be attributed to the decline in mental health in the general population (Black Dog Institute, 2020; Moreno et al., 2020; Pfefferbaum & North, 2020). However, our qualitative and quantitative data suggest that lower morale in 2020 is at least partly a function of the challenging personal and professional circumstances affecting many teachers and school leaders. The rapid shift from schooling-as-usual to learning from home caused a sharp intensification of teachers’ workloads. Simultaneously teachers created new ways to teach, adapted to unfamiliar tasks (such as new technologies) and then, upon the reopening of schools, found their students to be harder to engage, understandably challenging their efficacy.

Plausible explanations for why teachers struggled to engage students after they returned to school include: students having to re-adjust to the more rigid structures and controlled environment of school learning compared with learning from home; students distracted from learning by wider concerns than before the pandemic; students feeling fatigue as a result of more time in the classroom, a narrower curriculum focus on literacy and numeracy, and the absence of key extracurricular events such as school concerts and excursions; and teachers with lower morale, higher workloads and high levels of personal and professional stress struggling to put the same level of energy into their work as they had prior to the pandemic.

The last-minute nature of some communication to schools likely exacerbated effects on teachers’ morale and efficacy. In some instances, teachers and school leaders learned about policy changes from the media (at the same time as parents and students). As a result, teachers felt overlooked by governments, and they had no time to plan for coordinated school-based action in response to ongoing policy changes. Clear communication, which ensured schools were aware of changes before the broader community was notified, would have provided more support for school leaders and teachers during this difficult and demanding time for all.

When schools reopened, teachers were expected to rapidly return to ‘normal’, with great emphasis on ensuring students’ academic achievement, especially in the light of dire predictions about loss of learning (Brown et al., 2020; Goss & Sonnemann, 2020). The level of concern was clear in the state government’s investment of more than $330 million, in NSW alone, in a 2021 tutoring scheme designed to get students back where they should be. Similar investments were made elsewhere in Australia (Sonnemann & Hunter, 2021) and internationally (e.g. Burgess & Sievertsen, 2020). Some countries even considered extending the school day or shortening school holidays to help students ‘catch up’. These programs were rolled out rapidly, with ‘COVID tutors’ working in NSW schools within months of the announcement. However, research on past disasters that have affected schooling urges caution about placing too much pressure on academic outcomes, and not attending adequately to wellbeing (e.g. Long & Wong, 2012). While such studies typically draw attention to student wellbeing, our study highlights a much-needed focus on teacher wellbeing.

Work intensification, feelings of being expendable, rapid movement to radically different modes of teaching and a lack of recognition of the realities of their situation contributed to lower staff morale. Given the poor status of teacher wellbeing before COVID (Dabrowski, 2020), it is unlikely that the morale of the profession will simply lift on its own post COVID. While recent national investment in mental health (Hunt, 2021) is a positive start, additional support for teachers is required. In locations with higher numbers of COVID cases, communities and teachers are likely to also be grieving the loss of lives and dealing with the impact of more extended lockdowns. Teachers have not only felt undervalued for some time (Dabrowski, 2020; Dinham, 2013), they have been under enormous pressure and often felt overlooked during this collectively difficult and traumatising experience (Miller, 2020), where much was asked of them, and continues to be asked of them, with limited additional support. With ongoing ramifications of the COVID pandemic, more careful monitoring of teacher wellbeing is essential.

While we conducted a relatively small number of interviews (*n* = 18) in a state where there is a workforce of more than 30,000 primary school teachers, we attempted to mitigate this limitation by drawing the sample from a diverse range of NSW government schools and including the school-wide perspective of school leaders. Nonetheless, we acknowledge that teachers’ experiences, even within this sub-sample, did vary. Furthermore, our study focuses on the experiences of primary school teachers; research illuminating the experience of secondary school teachers is urgently needed.

## Conclusion

Rigorous empirical evidence is essential in understanding the effects of the COVID pandemic on teachers. Research on disruptions to schooling tends to focus on localised closures, often in relation to disasters (Convery et al., 2010; Kuntz et al., 2013; Whaley et al., 2017), rather than on the kinds of unprecedented system-wide closures caused by COVID and therefore have limited applicability to the current situation. Specific studies of the impact of COVID tend to concentrate on the experiences of students rather than teachers (Burkart et al., 2022; Drane et al., 2021; Grubic et al., 2020; Li et al., 2021). Our study offers a window into a crisis that has largely been obscured—how a very brief period (8 weeks) of system-wide school closure had primarily negative effects on teachers. This analysis raises questions about how we might better plan for a future of potential health crises and at least counter some of the negative effects on the morale and efficacy of teachers, into the future.

More than 168 million school children worldwide have been unable to attend school for more than a year due to school closures associated with COVID (UNICEF, 2021). By contrast, NSW schools were relatively unscathed, given a much shorter period of lockdown in 2020 than in many other contexts. Despite this relatively short lockdown, our study provides clear evidence of the impact of the COVID pandemic on teachers, signalling an urgent need for teacher wellbeing to be addressed here and in other systems.

Ongoing, rigorous and contextualised research into the effects of COVID on teachers is required. If we are to more fully understand the impact of school closures on teachers, such research should include: quantitative measures administered over time with the inclusion of multiple time points; robust comparable data obtained prior to the pandemic; and, clear articulation of the specific context and circumstances. In the meantime, systemic solutions that take account of the experiences of teachers during school closures, and consider the resources needed in all teaching contexts, should be developed and enacted without delay.

## Appendix 1

See Table 7.

**Table 7:** Means comparisons (original data, complete cases and imputed data)

Outcome	Group	Data	N	Mean		
				Term 1	Term 3	Term4
Engagement	2019	Original data	496	7.16	7.26	7.50
		Complete cases	294	7.10	7.24	7.47
		Imputed data	684	7.16	7.22	7.47
	2020	Original data	271	7.25	7.24	7.25
		Complete cases	189	7.26	7.17	7.32
		Imputed data	357	7.25	7.22	7.28
Instruction	2019	Original data	496	7.27	7.46	7.68
		Complete cases	294	7.29	7.42	7.71
		Imputed data	684	7.27	7.44	7.64
	2020	Original data	271	7.41	7.71	7.71
		Complete cases	189	7.42	7.65	7.77
		Imputed data	357	7.41	7.69	7.73
Management	2019	Original data	496	7.58	7.67	7.82
		Complete cases	294	7.52	7.67	7.82
		Imputed data	684	7.58	7.63	7.76
	2020	Original data	271	7.56	7.73	7.79
		Complete cases	189	7.51	7.70	7.88
		Imputed data	357	7.56	7.74	7.81
Morale	2019	Original data	496	4.04	4.13	4.29
		Complete cases	294	4.11	4.16	4.25
		Imputed data	684	4.04	4.11	4.27
	2020	Original data	270	4.14	4.08	4.04
		Complete cases	189	4.12	4.10	4.07
		Imputed data	357	4.14	4.09	4.06
Appraisal and recognition	2019	Original data	496	3.73	3.91	3.80
		Complete cases	294	3.84	3.92	3.78
		Imputed data	684	3.73	3.89	3.78
	2020	Original data	270	3.67	3.56	3.63
		Complete cases	189	3.66	3.59	3.59
		Imputed data	357	3.67	3.57	3.63

## Appendix 2

### Interview schedule

1. Can you tell me about the ‘learning from home’ arrangements implemented by your school this year?
2. How were you supported (by your school and/or the Department or others) in the transition to (and from) ‘learning from home’ arrangements?
3. What had been your experiences of using technologies to deliver lessons prior to the school shut down period?
4. What were the main challenges to you in delivering lessons via ‘learning from home’ arrangements?
  Possible prompt:
  - To what extent do you think your experiences are representative of other teachers at this school?
5. Were there any advantages to you in delivering lessons via ‘learning from home’?
  Possible prompts:
  - What worked well?
  - Is there anything that you tried/used/implemented during ‘learning from home’ arrangements that you will continue to use in your school/teaching?
  - To what extent do you think your experiences are representative of other teachers at this school?
6. What challenges did you hear about from students and families while they were ‘learning from home’?
7. Do you know of any particular advantages for your students or their families in students learning from home? Any specific disadvantages?
8. In your opinion, what impact has COVID-19 had on student achievement? How was this monitored or assessed?
9. Has student engagement altered in anyway as a result of COVID-19? If so, how?
10. What effect (if any) has the break from traditional schooling had on student wellbeing?
11. Were there any differences in the ways you approached teaching your class once school resumed full-time? If so, how did your teaching differ after the lockdown period from before the lockdown period?
12. Anything else you would like to tell us about your experience this year?

## Footnotes

1 Evacuation refers to the removal of all students from a school classroom due to emergency responses or behaviour escalation of one or more students. Suspension is a disciplinary consequence that prohibits students from attending school or school related activities for a period of time.
